# Unravelling the chemical design of spin-crossover nanoparticles based on iron(ii)–triazole coordination polymers: towards a control of the spin transition†
†Electronic supplementary information (ESI) available. See DOI: 10.1039/c5tc01093d



**DOI:** 10.1039/c5tc01093d

**Published:** 2015-06-22

**Authors:** Mónica Giménez-Marqués, M. Luisa García-Sanz de Larrea, Eugenio Coronado

**Affiliations:** a Instituto de Ciencia Molecular , Universidad de Valencia , c/ Catedrático José Beltrán, 2 , 46980 Paterna , Spain . Email: eugenio.coronado@uv.es; b Institut Lavoisier CNRS UMR 8180 , Université de Versailles St Quentin-en-Yvelines , 45, Av. des Etats Unis , 78035 Versailles Cedex , France

## Abstract

A systematic study of the key synthetic parameters that control the growth of spin-crossover (SCO) nanoparticles (NPs) using the reverse micelle technique has been undertaken in the system [Fe(Htrz)_2_(trz)](BF_4_)·H_2_O, (Htrz = 1,2,4-triazole). This has permitted us to modulate the physical properties of the NPs in a controlled and reproducible manner. In particular, a control over the size of the NPs (in the range 4 to 16 nm) has been achieved by varying the water to surfactant molar ratio. The consequences of this size variation on the cooperativity of the spin transition are discussed. Finally, this approach has been extended to the chemical alloy [Fe(Htrz)_2.95_(NH_2_trz)_0.05_](ClO_4_)_2_ in order to prepare NPs exhibiting a cooperative and hysteretic spin transition centred closer to room temperature.

## Abstract 2

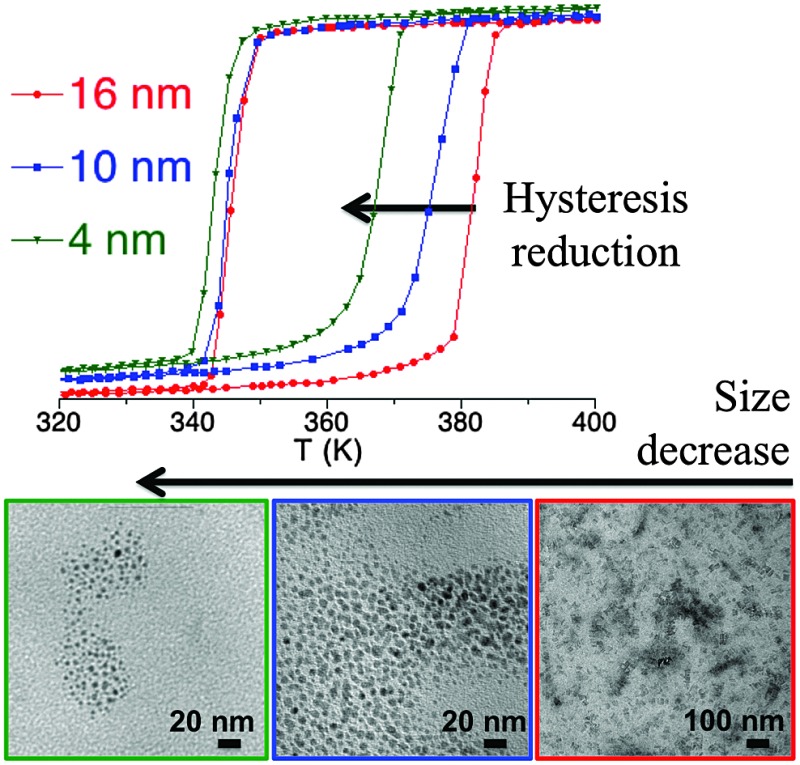
We present a systematic study of the key synthetic parameters that control the growth of Fe–triazole spin-crossover nanoparticles and the effect of this size modulation on the spin transition.

## 1. Introduction

Spin-crossover (SCO) materials represent one of the most promising classes of magnetic molecular materials to be used as molecular switches or memory storage devices.^1–3^ These coordination compounds undergo a change in their magnetic state upon application of an external stimulus such as temperature, light, pressure or electric field. Interestingly, other stimuli such as the physical sorption of gases have been recently proved to exert an influence over the SCO.^5^ To facilitate their integration into smaller and faster functional electronic devices a miniaturization down to the nanoscale is needed. With this aim, nanoparticles (NPs) of SCO complexes were obtained. The most convenient approach is based on the use of microemulsions to confine the growth of the coordination network (reverse-micelle approach). This method allowed us to obtain in 2007 NPs of the one-dimensional Fe^II^ polymeric family of general formula [Fe(Rtrz)_3_](A)_2_, (Rtrz = 4-substituted-4*H*-1,2,4-triazole, and A = monovalent anion) with an averaged size of *ca.* 10 nm displaying a very abrupt transition above room temperature with a wide thermal hysteresis of *ca.* 40 K, thus preserving the properties of the bulk material.^4^ These NPs where based on the particular system [Fe(Htrz)_2_(trz)](BF_4_)·H_2_O.^6^ In 2008 Létard *et al.* reported the preparation of NPs of [Fe(NH_2_trz)_3_](Br)_2_ (particle size of 60–200 nm) displaying a spin transition centred at room temperature.^7^ Since then, a large number of investigations have been devoted to the study of SCO NPs focusing mainly in two systems, the 1D [Fe(Rtrz)_3_](A)_2_ family^7–10^ and the family [Fe(pz){M(CN)_4_}], (pz = pyrazine, M = Ni, Pd, Pt) showing 2D or 3D structures.^11–13^ Actually, the control on the miniaturization of the SCO system has become one of the hot topics in the field. This is a challenging condition since the cooperativity needed to obtain a memory effect, highly depends on the particle size. Accordingly, a reduction of the NP volume may directly limit the cooperativity with the consequent lost of spin bistability. Indeed, the critical domain size at which hysteretic SCO behaviour may disappear is strongly dependent on the material.

Usually, the spin transition is accompanied by a change in the electron transport properties across the NPs. For example, when these NPs are contacted to two electrodes, a functional device displaying thermal hysteresis in the transport properties near room temperature is obtained. Such behaviour has been observed by our group either in devices formed by a single NP,^14^ as well as in devices formed by 2D assemblies of NPs.^15^ In the same vein, other groups have reported a similar electrical addressing in devices formed by assemblies of these SCO materials both at nanometric^16^ and micrometric^16,17^ scales. In addition, when these NPs are embedded in conducting polymers, the spin transition affords a large memory effect in the conductivity of the composite.^18^

Despite these promising results, progress in this area is strongly hindered by the difficulties encountered in the preparation of these NPs. In general, the sol–gel approach often presents reproducibility issues, mainly due to the large number of parameters associated with the processes of micelle formation and collision/coalescence of droplets. In fact, a synthetic protocol suitable for the preparation of SCO NPs in a precise and reproducible manner is still lacking. In this work we try to overcome this problem by improving the procedure used to prepare [Fe(Htrz)_2_(trz)_1_](BF_4_)·H_2_O (**1**) NPs. We will show that a systematic variation of the relevant parameters involved in the synthesis permit us to modulate the NP size (in the range 4–18 nm) in two families of SCO NPs, [Fe(Htrz)_2_(trz)_1_](BF_4_)·H_2_O (**1**) and [Fe(Htrz)_2.95_(NH2trz)_0.05_](ClO_4_)_2_ (**2**),^9^ with the consequent modulation of the SCO properties of these nanomaterials.

## 2. Results and discussion

### General reverse-micelle protocol for the preparation of [Fe(Htrz)_2_(trz)](BF_4_)·H_2_O (**1**) NPs

We develop here a general protocol that follows the original reverse micelle method previously established by our group^4^ with significant modifications in certain key parameters that have resulted to be crucial for improving its reproducibility and for a better understanding of the observed physical properties. We have performed this systematic study during the different steps of the synthesis, namely (i) micelle composition, (ii) microemulsion formation, (iii) micellar exchange, and (iv) NPs extraction.

#### (i) Micelle composition

This first step involves the preparation of two separate micellar dispersions incorporating the different reagents. At this stage the key parameters to be considered are the solvent and surfactant used, the ratio and concentration of reagents and finally the *ω*_0_ parameter (water to surfactant molar ratio). The type of solvent used is a relevant parameter to take into account since the particle size may be directly affected by the “solvent effect” since it can modulate the growth rate of the NP.^19^ For this method, *n*-octane has been used as the continuous phase, although *n*-hexane was also proved to be a possible solvent. Importantly, the solvent used to solubilize the precursors in the two separate micellar solutions is water. This is a modification from our previous method in which different solvents were used in each microemulsion (water to dissolve the iron salt and ethanol to dissolve the ligand). With this variation, the two micellar systems present an equivalent composition, thus minimizing possible side effects such as variation in density or polarity. Concerning the surfactant, the ionic AOT polymer has been maintained in the protocol. Compared with other non-ionic surfactants explored (Lauropal,^7,20a^ Triton X-100^10^), AOT presents a clear advantage: micelles formed by this ionic AOT afforded a smaller particle size for this system. Another example in this sense was presented by Bousseksou and co-workers with the use of an alcohol ethoxylate 15-S-3.^20*b*^

The use of behenic acid as the co-surfactant was proposed in the original synthetic procedure.^4^ Here we have investigated in detail its influence. Thus, three syntheses containing an excess of behenic acid, catalytic amounts of behenic acid, or no behenic acid were carried out. We observed that in the three cases the NPs presented similar particle sizes, while displaying very distinct colloidal stability with a gradual enhancement upon elimination of behenic acid. The conclusion was that colloidal stability is enhanced in the absence of this co-surfactant and, therefore, the synthesis of the NPs is feasible without using any co-surfactant. For these reasons, the behenic acid has been eliminated from the original synthetic procedure.

The appropriate molar ratio for the reagents Fe : Htrz has also been investigated. In a general procedure, different micellar systems with a fixed concentration of Fe^2+^ ([Fe] = 1 M) and variable amounts of Htrz have been used to obtain the corresponding molar ratios 1 : 2.5, 1 : 3 and 1 : 5. The resulting suspensions were analyzed by DLS and the results are shown in Table 1. In the case of the triazole deficient system (molar ratio 1 : 2.5), a precipitate is formed during the intermicellar exchange, while NPs presenting sizes >50 nm remain stable in the suspension. When an excess of triazole is used instead (molar ratio 1 : 5) the polydispersity index (PdI) dramatically increases and NPs of 8 and 42 nm are obtained simultaneously. Finally, for the stoichiometric 1 : 3 molar ratio, NPs of 15 nm with a narrow distribution have been obtained. These results clearly indicate that the appropriate relation of reagents is the stoichiometric 1 : 3 molar ratio.

**Table 1:** Size for the NPs synthesized with different Fe : Htrz molar ratios

[Fe]	[Htrz]	Size (DLS based)
1	2.5	>50 nm and >1 μm
1	3	15 nm
1	5	8 and 42 nm

The optimal concentration of reagents inside the micelle was also investigated. Several studies suggest that by increasing this concentration, the size of the NP is reduced with a low polydispersity.^19,21^ The highest concentration that permits a correct stabilization of the micellar solution is considered to be the optimal Fe^+2^ concentration. Different Fe^+2^ concentrations – [Fe] = 0.5, 1 and 1.5 M – have been tested by changing the quantity of Fe(BF_4_)_2_·6H_2_O while maintaining constant the water volume (0.3 mL) and the surfactant (1 g), and using stoichiometric amounts of Htrz for each case. For [Fe] < 0.5 M, a brown colour appears upon mixture of the two micellar solutions, indicating a partial or complete oxidation of the Fe^+2^. For [Fe] > 1.5 M, stoichiometric amounts of Htrz ([Htrz] > 4.5 M) are not soluble in 0.3 mL of water. DLS analysis together with elemental analysis (EA) for the three different Fe^+2^ concentrations are shown in Table S1 (ESI†). From these data we can conclude that a Fe^+2^ concentration 1 M presents some advantages with respect to the other concentrations. This concentration permits the formation of NPs with a reduced size compared with [Fe] = 0.5 M, and the effective and rapid micelle formation as compared with [Fe] = 1.5 M. Consequently, for this system the most suitable concentrations of Fe and Htrz are 1 M and 3 M, respectively. Finally, the role of the *ω*_0_ parameter requires a more complex investigation that will be discussed in the following section.

#### (ii) Microemulsion formation

The micelle formation occurs upon vigorous stirring at room temperature enabling the mixture of the different components in the micellar system and thus resulting in the formation of the micelles in a homogenous medium. In this step the key factor is the time required for stabilizing the micelle upon stirring. This stabilization can be easily detected since a visually clear and translucent homogenous solution suggests the formation of the micelles. The time required to obtain a homogenous optically isotropic and thermodynamically stable microemulsion depends on the ratio water/surfactant, *ω*_0_ but also on the nature of the micelle. For instance, for *ω*_0_ = 7, the Fe microemulsion ([Fe] = 1 M) is stabilized in *ca.* 30 minutes, while it takes *ca.* 3 min to stabilize the triazole microemulsion ([Htrz] = 3 M). This difference may be attributed to the different pH of both micellar systems (1–2 for the Fe microemulsion and 6–7 for the triazole microemulsion). We have investigated the time for micelle stabilization in several reactions using different concentrations (0.1 < [Fe] < 1.5 M, and 0.3 < [Htrz] < 3 M). From these studies, several considerations can be extracted: (i) for Fe microemulsions, the time required to stabilize the microemulsion shows a linear dependence with the concentration in the 0.1–1.5 M range (Fig. S2, ESI†); (ii) for Htrz microemulsions the stabilization time is very rapid and remains practically the same in the 0.3–3 M range.

#### (iii) Micellar exchange

This step comprises the combination upon stirring at room temperature of the two separated microemulsions. The reaction time has been reduced from 4 h to 30 min, for which completion of the intermicellar exchange is already reached. Further stirring has been proved to promote no effect on the final NP size.

#### (iv) NPs extraction

This last part has also been modified in order to better control when the nanocrystal growth is stopped, but also to improve the elimination of the surfactant in excess. Thus, destabilization of the micelles is done upon addition of acetone, which promotes the precipitation of the NPs and allows their extraction by centrifugation. Further washing with acetone improves the elimination of the excess of reagents. By using this new method of precipitation/extraction, the excess of surfactant in the final NPs has been highly reduced. Still, the elimination of the excess of surfactant is not complete. This may be related to the ionic nature of the AOT that favours the anionic exchange with the BF_4_^–^. As a result, part of the AOT strongly interacts with the NP hindering its total elimination.

### Influence of the water to surfactant molar ratio (*ω*_0_) on the size of the [Fe(Htrz)_2_(trz)](BF_4_)·H_2_O (**1**) NPs

**Fig. 1:**
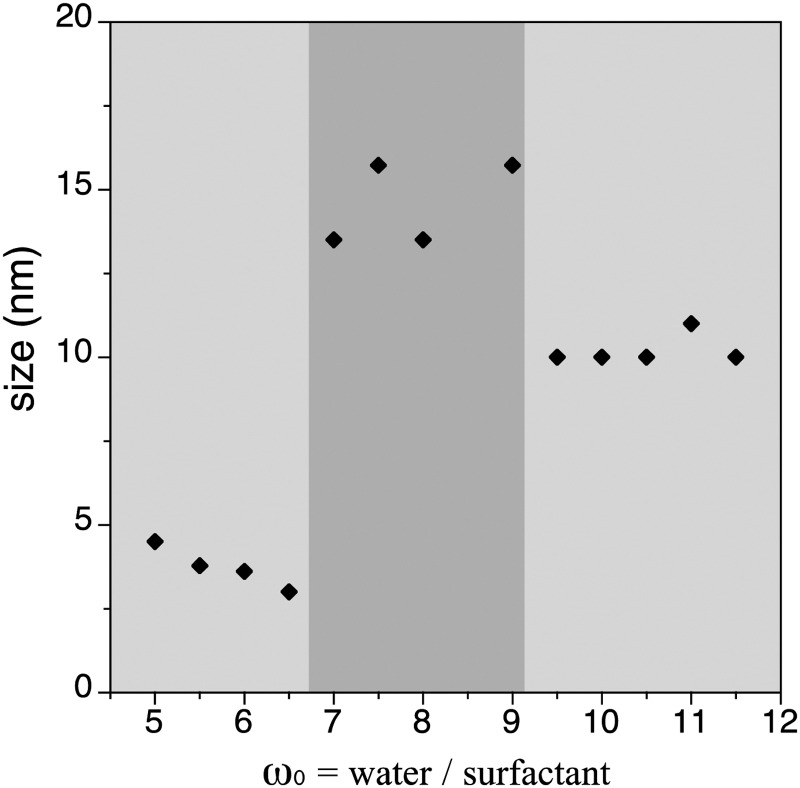
Representation of the NP size of **1.1–1.13** as a function of the *ω*_0_ parameter.

Recent studies in the field of SCO have focused on the effective controlled miniaturization of bistable NPs using mainly two approaches that are based either on the modification of the *ω*_0_ parameter,^9^ or the change in concentration of the metal salt.^11,22^ In particular, the influence of the parameter *ω*_0_ on the size of NPs obtained by micellar systems has been at the origin of intense debate in the past decades.^23^ In fact, micelles act as chemical reactors and, in principle, it is possible to influence the NP size by fine-tuning the micellar size. General conclusions claim that an increase of the *ω*_0_ parameter produces an increase of the droplets and therefore an increase of the NP size, although different results can be obtained depending on the nature of the system under study. For **1**, this effect was already studied by our group in a preliminary work.^9^ The general trend (decrease of the size upon decrease of water to surfactant molar ratio) was observed for two different sizes. In particular, a decrease in *ω*_0_ from 15.8 to 10.0 ended up with a decrease in the size of the NPs from 11 ± 5 nm to 6 ± 3 nm. Unfortunately, further decrease of *ω*_0_ down to 7.4 led to the precipitation of micrometric particles, impeding to draw a definite conclusion. With these results in mind and considering the above-mentioned synthetic modifications, we have undertaken an exhaustive analysis to establish the effect of the *ω*_0_ on the final particle size. To ensure a comparable study, all micellar compositions have been systematically preserved (see Table 2), whereas AOT content has been subsequently modified. Thus, a series of NPs **1.1–1.13** based in (**1**) has been synthesized with a progressive increase of *ω*_0_. The *ω*_0_ range of study was determined following several considerations: (i) for NPs obtained with *ω*_0_ < 5.0 a rapid precipitation occurs during the micellar exchange; (ii) microemulsions formed with *ω*_0_ > 11.5 are only stabilized after long times (*i.e.* extremely large time for micellar formation >5 hours). Consequently, the study has been centred in the 5.0 < *ω*_0_ < 11.5 range. Table 3 summarizes the NPs synthesized with the different *ω*_0_ values showing their corresponding NP size obtained by direct measuring the suspension of the as synthesized NPs in *n*-octane by DLS. A representation of the particle size as a function of *ω*_0_ is depicted in Fig. 1, where three different zones with clearly distinct size tendencies can be distinguished. The first region corresponds to samples **1.1–1.4**, synthesized in the 5 < *ω*_0_ < 6.5 range, for which an average particle size of *ca.* 4 ± 2 nm is obtained. Upon further increasing the *ω*_0_ parameter (*i.e.* decreasing the AOT content), a clear increase of the NP size is observed for samples **1.5–1.8**, synthesized in the 7 < *ω*_0_ < 9 range, which present an average size of *ca.* 15 ± 5 nm. Finally, successive increase of *ω*_0_ from 9.0 to 11.5 in samples **1.9–1.13** affords a new series in which the same particle size is constantly obtained (*ca.* 10 ± 4 nm).

**Table 2:** General parameters used in all the synthesis to study the *ω*_0_ effect

[Fe]	[Htrz]	*V*_H_2_O_ (mL)	*V*_n-octane_ (mL)	mol AOT
1 M	3 M	0.3	10	Variable

**Table 3:** Samples obtained as a function of the *ω*_0_ parameter and corresponding size distribution measured by DLS (number-based)

Sample	*ω*_0_	Size (nm)	Sample	*ω*_0_	Size (nm)
—	<5.0	>1 μm	**1.7**	8.0	14 ± 5
**1.1**	5.0	5 ± 2	**1.8**	9.0	16 ± 5
**1.2**	5.5	4 ± 2	**1.9**	9.5	10 ± 4
**1.3**	6.0	4 ± 3	**1.10**	10.0	10 ± 3
**1.4**	6.5	3 ± 2	**1.11**	10.5	10 ± 5
**1.5**	7.0	14 ± 4	**1.12**	11.0	11 ± 4
**1.6**	7.5	16 ± 4	**1.13**	11.5	10 ± 4

**Fig. 2:**
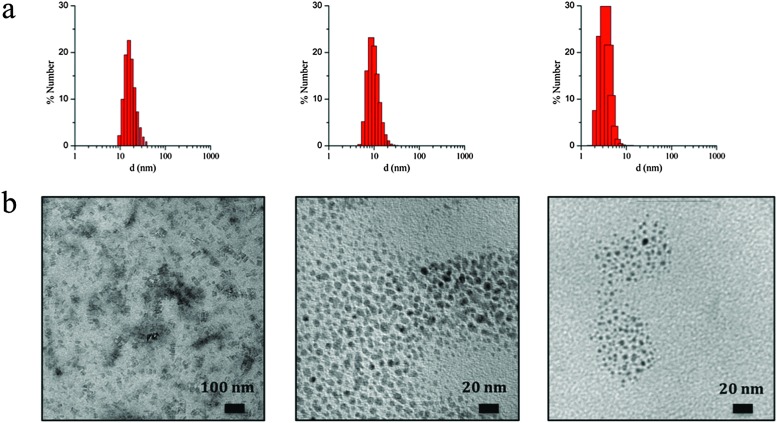
(a) Number-based particle size distribution for the colloidal suspension **1.6** (left), **1.10** (middle) and **1.2** (right) obtained by DLS analysis. (b) TEM images of the same NPs displaying sizes of 16 nm (left), 10 nm (middle) and 4 nm (right) deposited by drop casting on a carbon coated copper grid (left) or placing an ultrathin-section of the dispersed colloids using cryo-microtomy (middle and right).

**Fig. 3:**
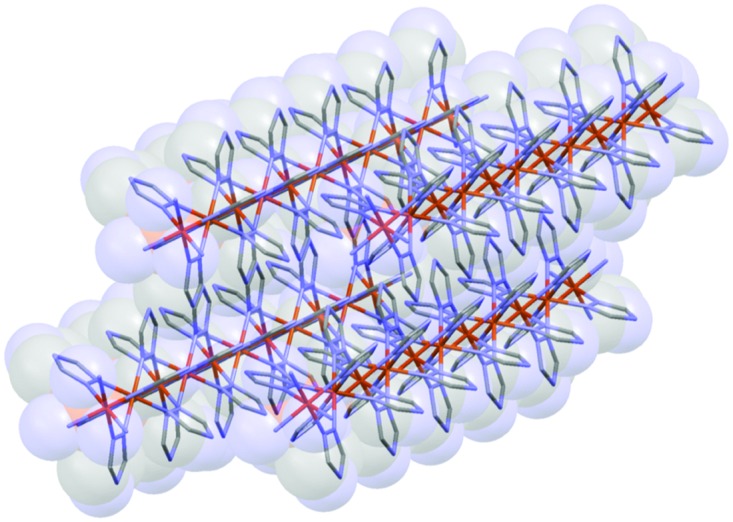
Cartoon representation of the most likely composition of the 4 nm NPs.

The observed irregular size trend upon decrease of *ω*_0_ differs from the anticipated linear size dependence. Interestingly, this tendency has been already detected in different systems^24^ and may be explained considering the three different scenarios existing inside the micelles at each given *ω*_0_ range:^23*e*^ (i) at low *ω*_0_ (5 < *ω*_0_ < 6.5), the water inside the reverse micelles (low water content) may be considered “bound”, since there is insufficient water available to solvate both the surfactant head group and the counterion. With the water “bound”, the micelle interface is considered as “rigid”, lowering intermicellar exchange and therefore decreasing the growth rates. Therefore, this ends with small NPs due to their difficulty in growing; (ii) as *ω*_0_ is raised (7 < *ω*_0_ < 9), the micelle interface becomes more fluid due to the presence of more water molecules, so the rate of growth increases until a given *ω*_0_ value, promoting an increase of the particle size;^25,26^ (iii) at this point, upon further increase of the *ω*_0_ parameter (9 < *ω*_0_ < 11.5) the micelles present an extra content of water, which merely dilutes the reagents and decreases the reaction rates, leading to a decrease in the particle size. A thorough characterization of three selected samples of SCO-NPs representative of each group of sizes has been developed: samples **1.6** (16 nm), **1.10** (10 nm) and **1.2** (4 nm). The particle size of all NPs based in [Fe(Htrz)_2_(trz)](BF_4_)·H_2_O was established by DLS with polydispersity indices consistent with monodispersed samples (PdI < 0.2) and then supported by TEM analysis (see Fig. 2). The amount of AOT associated with the NPs was determined by elemental analysis (Fig. S3, ESI†). X-ray powder diffraction (XRPD) patterns were obtained on microcrystalline powdered samples, to confirm that the crystalline structure of **1** was maintained (Fig. S4, ESI†). Note that a peak broadening is observed in comparison with the bulk material, consistent with the reduced particle size. Finally, an estimation of the number of Fe^2+^ atoms that contains each single NP can be done by considering the size and the structural parameters (unit cell: 17.35 Å, 7.33 Å, 9.19 Å, 90°, 90°, 90°; space group *Pnma*; *Z* = 4)^27^ of the NPs. Importantly, due to the dimensionality of the system, the morphology of the NPs plays an important role in this estimation since the number of Fe^2+^ atoms strongly depend on the direction of growth. The reduced size and hybrid nature of the NPs together with the presence of the organic coating makes difficult an analysis of the NP shape by TEM. Nevertheless, we have successfully observed by HR-TEM the two different NP orientations (parallel or standing) after sample deposition by drop casting in sample **1.16** (*ca.* 16 nm) (see Fig. S5, ESI†). This is also in agreement with the morphology observed in self-assembled monolayers of NPs in our recent work, in which rod-like shape arrangement have been determined using high-angle annular dark field scanning transmission electron microscopy (STEM-HAADF).^15^ In addition, we have also investigated this morphology by means of Atomic Force Microscopy (AFM) (see Fig. S6, ESI†). This assortment of techniques provided images evidencing that these NPs are not spherical as initially thought but possess an elongated shape, with one dimension approximately twice the other two. The larger direction could in principle be assigned to the *b* parameter, which corresponds to the direction in which the cationic chains [Fe(Htrz)_2_(trz)]^+^ are aligned, although to confirm this point a crystal indexation by single crystal diffraction is needed. Table 4 summarizes the number of Fe^2+^ atoms in relation with the NP size, and Fig. 3 illustrates the most likely composition of the 4 nm NPs.

**Table 4:** Number of Fe^II^ atoms in relation with the NP size for samples **1.6**, **1.10** and **1.2**

Sample	Size (nm)	Number of Fe^2+^ atoms
**1.6**	16	3960
**1.10**	10	840
**1.2**	4	40

### Influence of the size on the spin transition of [Fe(Htrz)_2_(trz)](BF_4_)·H_2_O (**1**) NPs

**Fig. 4:**
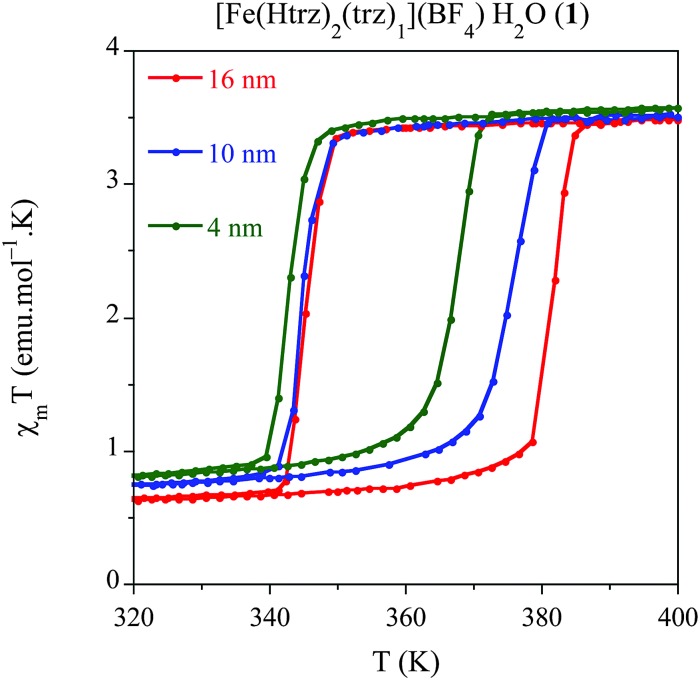
Temperature dependence of the molar magnetic susceptibility temperature product (*χ*_M_ *T*) for samples **1.6** (16 nm), **1.10** (10 nm) and **1.2** (4 nm) after several heating–cooling modes.

Once established the best synthetic parameters and the *ω*_0_ size dependence for this system, the next step consists of studying how the size variation affects the spin transition. For this purpose, solid dried samples with sizes 16 nm (**1.6**), 10 nm (**1.10**) and 4 nm (**1.2**) have been used for magnetic measurements. The thermal dependence of *χ*_M_ *T* for the three samples is depicted in Fig. 4, where *χ*_M_ is the molar magnetic susceptibility (in emu mol^–1^ K). Thermal spin transitions are characterized by *T*↑1/2 and *T*↓1/2 which are defined as the temperatures for which 50% of both the LS and HS Fe^2+^ centres are present in the sample for the heating and cooling modes, respectively. Finally, Δ*T* represents the hysteresis width that appears in all the spin transitions. All these parameters are summarized in Table 5. The largest NPs (*ca.* 16 nm) exhibit an abrupt spin transition centred above room temperature with a large hysteresis of 38 K (*T*↑1/2 = 379 K and *T*↓1/2 = 341 K), which is comparable to that of the bulk material (*T*↑1/2 = 385 K and *T*↓1/2 = 345 K). This behaviour is reproducible over successive thermal cycles. A relatively large HS remnant fraction of 20% (higher than that found in the bulk) remains at low temperatures, which is indeed typical for these nanostructured chain compounds. This characteristic HS remnant fraction may be ascribed to the different coordination environment of the terminal Fe in the chains, which may be coordinated to oxygen atoms from water ligands instead of the nitrogen atoms of the triazole ligand. Therefore, the terminal Fe in the chain presents a lower ligand field strength, which favours the HS state in all range of temperatures. In nanostructured materials, the number of terminal Fe is larger than in the bulk, and consequently the remnant fraction is also larger. When the particle size is reduced to 10 nm, the magnetic behaviour starts to deviate from the bulk. A hysteresis of 31 K (*T*↑1/2 = 374 K and *T*↓1/2 = 343 K) is found in this case, with a similar remnant HS residue (∼20%) whereas the sharpness of the hysteresis is maintained. Magnetic measurements of the smallest particles obtained (*ca.* 4 nm) show a reduced hysteresis loop of 24 K (*T*↑1/2 = 367 K and *T*↓1/2 = 343 K), which corresponds to a 40% reduction of the hysteresis found in the macroscopic material. The occurrence of a thermal hysteresis of 24 K in NPs as small as 4 nm is highly remarkable. This behaviour may be attributed to several facts: (i) NPs of **1** are based in a 1D coordination polymer and therefore may result less affected by the size reduction as compared with other 3D-based systems, which undergo a fast decrease of the hysteresis width upon size reduction.^11^ Since the cooperativity is directly dependent on the elastic forces present in the SCO material, one should expect that in our chain polymer the dominant elastic forces occur along the chain direction (*i.e.*, they will have a strong 1D character). With this assumption, the size reduction effects in our anisotropic chain system should depend on the length of the chains (*i.e.*, on *r*) whereas in isotropic 3D systems they should depend on the volume of the NP (*i.e.*, on *r* ^3^); (ii) in addition, this structural anisotropy is enhanced by the shape anisotropy of the NPs (rod-like shape).^9,14b^ This effect should also contribute to maintain the cooperativity along the Fe chains since, even for a very small average size the length of the chains is still above a critical value (according to our calculations, the 4 nm NPs are estimated to contain 4 chains of *ca.* 10 Fe^2+^ atoms in length). The HS residue is of *ca.* 23%, which is slightly higher than the one observed in the larger NPs, as expected. In addition, the sharpness of the transition is significantly maintained.

**Table 5:** Physical characteristics of the thermal spin transition *T*↑1/2 and *T*↓1/2 and particle sizes

Sample	Size (nm)	*T* ↑ 1/2	*T* ↓ 1/2	Δ*T*	% HS
**1.6**	16	379	341	38	20
**1.10**	10	374	343	31	20
**1.2**	4	367	343	24	23

In summary, this study shows that the thermal hysteresis is reduced with the particle size, as expected, although it also evidences the strong effective cooperativity exhibited by this particular SCO system, which exhibits a thermal hysteresis even for the smallest particles (*ca.* 4 nm). Furthermore, this study also shows that the transition temperature only changes in the heating mode whereas it remains stable in the cooling mode. A theoretical explanation of this behaviour is in progress.

### Influence of the size on the spin transition in the chemical alloy [Fe(Htrz)_2.95_(NH_2_trz)_0.05_](ClO_4_)_2_ (**2**) NPs

**Fig. 5:**
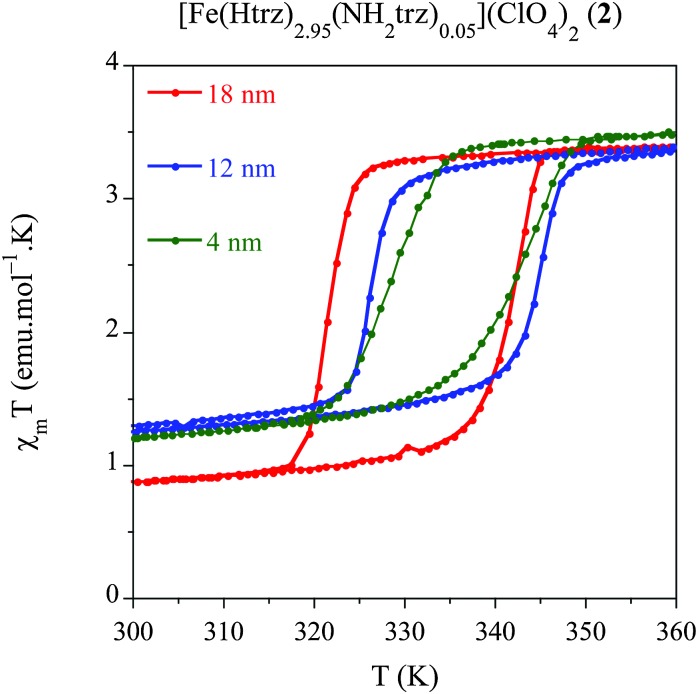
Temperature dependence of the molar magnetic susceptibility temperature product (*χ*_M_ *T*) for samples **2.1** (18 nm), **2.2** (12 nm) and **2.3** (4 nm) after several heating–cooling modes.

We have developed a comparative study in an additional related compound, [Fe(Htrz)_2.95_(NH_2_trz)_0.05_](ClO_4_)_2_ (**2**) using our modified protocol with the aim of studying the effect of the particle size, while shifting the spin transition towards room temperature. This polymer presents a particular chemical composition, which consists in a partial ligand substitution (<2%) of the triazole ligand by its derivative 4-amino-triazole (NH_2_trz) ligand. As previously reported by our group,^9^ this chemical modification directly affects the spin transition, which progressively moves towards lower temperatures. In particular, this alloy has been selected since it displays a spin transition centred at temperatures closer to room temperature with preservation of a strong cooperative behaviour. In a similar study than that developed for system **1**, we have obtained NPs of the alloy compound **2** with sizes 18 nm (**2.1**), 12 nm (**2.2**) and 4 nm (**2.3**) by selecting the appropriate *ω*_0_ value (see Experimental section). Magnetic measurements performed on dried samples of the different NPs are shown in Fig. 5 whereas all magnetic parameters are summarized in Table 6. We have represented the magnetic moment as a function of the temperature of these three samples for a direct comparison of the hysteresis loops (see Fig. 5). The largest NPs **2.1** (*ca.* 18 nm) display an abrupt spin transition centred closer to room temperature with a hysteresis of 20 K (*T*↑1/2 = 341 K and *T*↓1/2 = 321 K) and a HS remnant fraction of 25%. This behaviour is reproducible over successive thermal cycles. NPs **2.2** presenting 12 nm of size, display a reduced hysteresis of 18 K (*T*↑1/2 = 344 K and *T*↓1/2 = 326 K), with a larger remnant HS residue (∼35%). Finally, the smallest particles **2.3** (*ca.* 4 nm) show a reduced hysteresis loop of 13 K (*T*↑1/2 = 341 K and *T*↓1/2 = 328 K) with a similar HS residue (∼34%). This result has demonstrated that, using the synthetic protocol developed in this work, SCO NPs of various sizes exhibiting a spin transition in the vicinity of room temperature can be prepared. These NPs exhibit cooperative effects (thermal hysteresis) even for the smallest NPs of 4 nm.

**Table 6:** Physical characteristics of the thermal spin transition *T*↑1/2 and *T*↓1/2 and particle sizes

Sample	Size (nm)	*T* ↑ 1/2	*T* ↓ 1/2	Δ*T*	% HS
**2.1**	18	341	321	20	25
**2.2**	12	344	326	18	35
**2.3**	4	341	328	13	34

## 3. Conclusions

Following a reverse micelle approach, we have developed a systematic and reproducible protocol to prepare SCO NPs based on the system [Fe(Htrz)_2_(trz)](BF_4_)·H_2_O. A careful evaluation of the most relevant parameters that play a crucial role in the final physico-chemical properties of the NPs has been undertaken. The key parameter has shown to be the water to surfactant molar ratio (*ω*_0_) as it controls the size of the NPs.

The effect of reducing the size of the NP on the spin transition (from 18 nm to 4 nm) has been explored in two related materials, [Fe(Htrz)_2_(trz)](BF_4_)·H_2_O (**1**) and [Fe(Htrz)_2.95_(NH_2_trz)_0.05_](ClO_4_)_2_ (**2**). The second material has been studied with the aim of shifting the SCO phenomenon towards room temperature. Importantly, for the different sizes investigated, the NPs exhibit the characteristic abruptness in the spin transition, with a significant decrease of the hysteresis width by progressive reduction in the size. Thus, relatively large hysteresis have been preserved even for NPs of only 4 nm (with values of 24 K and 13 K for compounds **1** and **2**, respectively). This may be attributed to the strong cooperativity and low dimensionality of this particular family of compounds. In this sense, this family of NPs is probably the most promising candidate for the implementation of SCO-NPs in molecular-based nanodevices.

## 4. Experimental section

### Synthesis of [Fe(Htrz)_2_(trz)](BF_4_)·H_2_O (**1**) and [Fe(Htrz)_2.95_(NH_2_trz)_0.05_](ClO_4_)_2_ (**2**) NPs

**1**: an aqueous solution of Fe(BF_4_)_2_·6H_2_O (0.3 mL, 1 M) is added to a previously prepared solution of the surfactant dioctyl sulfosuccinate sodium salt, NaAOT (1.00 g for **1.6**, 0.74 g for **1.10** and 1.35 g for **1.2**) in *n*-octane (10 mL). Then, the micellar solution is stirred at room temperature until a thermodynamically stable microemulsion is formed (*ca.* 30 minutes). Similarly, an aqueous solution of Htrz (0.3 mL, 3 M) ligand is added to a solution of NaAOT (1.00 g for **1.6**, 0.74 g for **1.10** and 1.35 g for **1.2**) in *n*-octane (10 mL) to form also a microemulsion. Once micellar solutions are stable, the two microemulsions are mixed at room temperature under Ar atmosphere and stirred 30 min to ensure the micellar exchange. The NPs are obtained by precipitation upon addition of acetone to destabilize the microemulsion and collected by centrifugation (12 000 rpm, 10 minutes), after several washing cycles with portions of acetone (×3) and ethanol (×3) to remove excess of surfactant. Finally, the powdered samples are dried under vacuum for 2 h to remove excess of solvent.

**2**: an aqueous solution of Fe(ClO_4_)_2_·6H_2_O (0.3 mL, 1 M) with *ac*. 10 mg of ascorbic acid is added to a previously prepared solution of NaAOT in 10 mL of *n*-octane (1.00 g for **2.1**, 0.74 g for **2.2** and 1.35 g for **2.3**) under vigorous stirring. Similarly, a mixture of Htrz (61.1 mg) and NH_2_trz (1.3 mg) ligands are dissolved in 0.3 mL of water and added to a solution of NaAOT in 10 mL of *n*-octane (1.00 g for **2.1**, 0.74 g for **2.2** and 1.35 g for **2.3**) also under vigorous stirring. Once the two microemulsions become stable, they are mixed and stirred for 15 min at room temperature under Ar atmosphere. The NPs are collected following the same procedure described for **1**.

### Sample characterization

TEM images were obtained from a JEOL JEM 1010 microscope (100 kV). Sample preparation was specifically adjusted for each sample, by placing a drop of the NPs suspended in a solvent on a carbon coated copper grid or an ultrathin-section of the dispersed colloids using cryo-microtomy methods. The NP size distribution was determined in suspensions by DLS using a Zetasizer ZS (Malvern Instruments, UK) and with TEM images analysis after their deposition. Phase purity of all samples was established by PXRD. Polycrystalline samples of the NPs were lightly ground in an agate mortar and pestle and filled into a 1 mm borosilicate capillary. Data were collected at room temperature in the 2*θ* range 5–35° on an Empyrean PANalytical powder diffractometer, using Cu K_α_ radiation. Carbon, nitrogen, hydrogen and sulfur contents were determined by microanalytical procedures using an EA 1110 CHNS-O elemental analyzer from CE Instruments. Magnetic susceptibility measurements were performed on single-phased polycrystalline samples of **1.6**, **1.10**, **1.2** and **2.1**, **2.2** and **2.3** with a Quantum Design MPMS-XL-5 SQUID susceptometer. The susceptibility data were corrected from the diamagnetic contributions as deduced by using Pascal's constant tables. The data were collected in the range 300–400 K upon recording several heating–cooling modes at a constant rate with an applied field of 0.1 T. AFM images were measured in a Nanoscope IIIa AFM (Veeco) in tapping mode for a NP suspension in *n*-octane (0.1 mg mL^–1^) (sample **1.6**, 16 nm) deposited by drop casting on native SiO_2_.
