# Supplementary material for: Unravelling the chemical design of spin-crossover nanoparticles based on iron(ii)–triazole coordination polymers: towards a control of the spin transition
Source: J Mater Chem C Mater. 2015 Jun 22;3(30):7946–53. doi: 10.1039/c5tc01093d (PMC4894072; doi:10.1039/c5tc01093d)
Supplement: Supplementary file 1 [file TC-003-C5TC01093D-s001.pdf]

Electronic Supplementary Material (ESI) for Journal of Materials Chemistry C.
This journal is © The Royal Society of Chemistry 2015

Unravelling synthetic key parameters for the design of spin-crossover nanoparticles based on iron(II)-triazole coordination polymers: towards a control of the spin transition

Mónica Giménez-Marqués, M. Luisa García-Sanz de Larrea and Eugenio Coronado*.

Instituto de Ciencia Molecular, Universidad de Valencia, c/ Catedrático José Beltrán, 2, 46980 Paterna, Spain.

Supporting Information

General reverse-micelle protocol for the preparation of [Fe(Htrz)$_2$(trz)](BF$_4$)·H$_2$O (1) NPs.

S1. Size analysis and elemental analysis.

S2. Stabilization time for the Fe^{II} microemulsions as a function of the metal concentration (0.1–1.5 M).

S3. Images of microemulsion stabilization.

Controlling size in [Fe(Htrz)$_2$(trz)](BF$_4$)·H$_2$O NPs: size effect of ω_0 variation.

S4. Size analysis and elemental analysis.

S5. XRPD analysis.

S6. HR-TEM analysis.

S7. AFM analysis.

References

General reverse-micelle protocol for the preparation of $[Fe(Htrz)_2(trz)](BF_4)\cdot H_2O$ (1) NPs.

Table S1. Size (DLS-based) and elemental analysis for NPs synthesized with different [Fe].

[Fe]	Size (nm)	Elemental analysis	C [%]	N [%]	H [%]	S [%]	MW
0.5	25	experimental	33.57	22.39	4.49	3.21	
		$[Fe(Htrz)_2(trz)](BF_4)\cdot H_2O\cdot(AOT)_{0.6}$	34.18	19.93	4.97	3.04	635,79
1	15	experimental	19.87	32.31	3.13	0.22	
		$[Fe(Htrz)_2(trz)](BF_4)\cdot H_2O\cdot(AOT)_{0.02}$	20.51	33.64	2.62	0.17	374,73
1.5	11	experimental	19.08	30.85	2.39	0.41	
		$[Fe(Htrz)_2(trz)](BF_4)\cdot H_2O\cdot(AOT)_{0.04}$	21.29	32.86	2.75	0.33	383,62

Figure S2. Representation of the stabilization time for the Fe^{II} microemulsions as a function of the metal concentration (0.1–1.5 M) with $\omega_0 = 5$ (the black line serves as a reference to linearity).

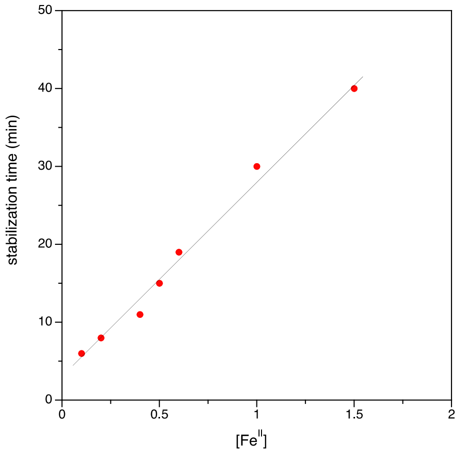

Figure S3. Pictures of the two micellar solutions before (a) and after (b) micelle stabilization upon stirring showing the difference in transparency.

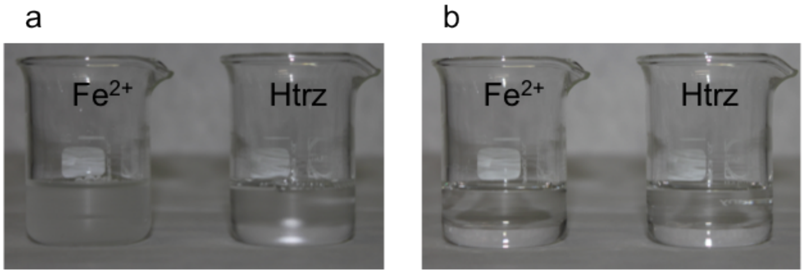

Controlling size in $[Fe(Htrz)_2(trz)](BF_4)$ NPs: size effect of ω_0 variation.

Table S4. Table of size and elemental analysis for samples **1.16**, **1.10** and **1.2**.

Sample	Size (nm)	Elemental analysis	C [%]	N [%]	H [%]	S [%]	MW
**1.16**	16	experimental	25.59	28.93	3.48	1.77	
**1.10**	10	experimental	26.24	28.82	3.38	1.66	

**1.2**	4	experimental	29.63	26.13	3.99	2.54	
Theoretical		$[Fe(Htrz)_2(trz)](BF_4)\cdot H_2O\cdot(AOT)_{0.2}$	26,74	28,06	6,68	0,71	449,25
Theoretical		$[Fe(Htrz)_2(trz)](BF_4)\cdot H_2O\cdot(AOT)_{0.3}$	28,87	25,25	4,06	1,93	499,21

Figure S5. X-ray powder diffraction (XRPD) patterns of samples **1.6** (16 nm), **1.10** (10 nm) and **1.2** (4 nm) obtained on powdered samples. The patterns of the NPs bearing different sizes are compared with the experimental pattern of microcrystalline powder of $[Fe(Htrz)_2(trz)](BF_4)\cdot H_2O$ (**1**) obtained as bulk.[1]

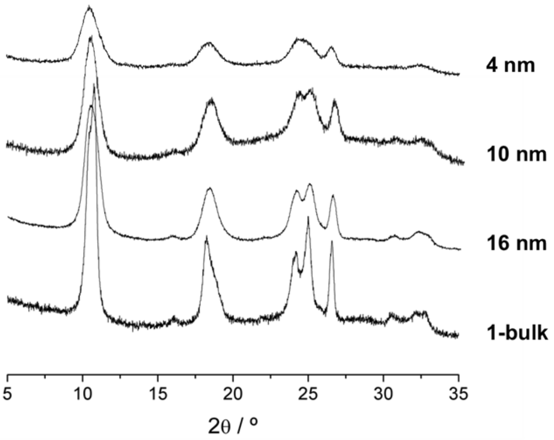

Figure S6. HR-TEM image of sample **1a** (*ca.* 16 nm) showing the two different orientations (parallel or standing) after sample deposition by drop casting. These two different orientations have been also observed by some of us in self-assembled monolayers of these NPs, using high-angle annular dark field scanning transmission electron microscopy (STEM-HAADF).[2]

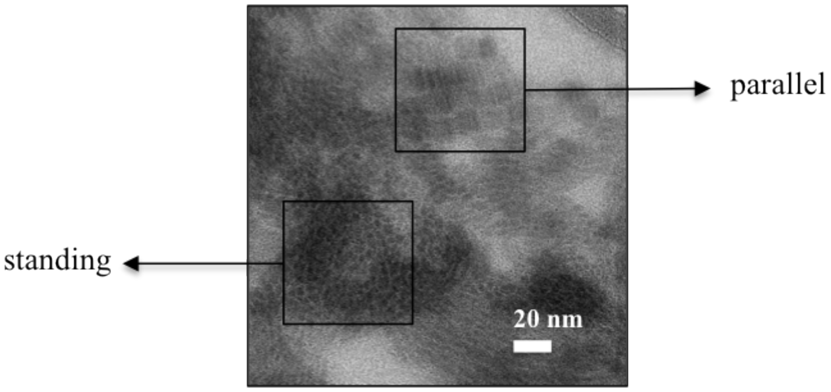

Figure S7. AFM (topography (a) and phase (b)) images measured in tapping mode for SCO-NPs (sample **1.16**, 16 nm) deposited by drop casting on native SiO_2.

Silicon substrate cleaning process. Substrates of *ca.* 1 cm^2 were sonicated for 10 minutes in a freshly-prepared $H_2O_2:NH_4OH:H_2O$ (1:1:2) solution (x3 times). The substrates are then rinsed with mili-Q water, sonicated 5 minutes in mili-Q water (x2 times) and finally dried under a N_2 stream (*ultrasonic cleaner*: BRANASONIC MTH-5510 ultrasonic cleaner, power 185 W).

Drop casting deposition: A drop of the SCO-NP suspension is dropped on top of the substrate and left unperturbed for 30 second. Then, the substrate is rinsed with *n*-octane and dried under a N_2 stream.

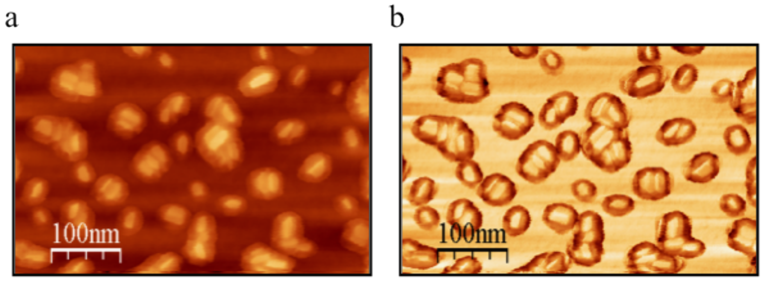

References

[1] A. Grosjean, P. Négrier, P. Bordet, C. Etrillard, D. Mondieig, S. Pechev, E. Lebraud, J.-F. Létard, P. Guionneau, *Chem. Commun*. **2011**, *47*, 12382.
[2] J. Dugay , M. Giménez-Marqués , T. Kozlova , H. W. Zandbergen , E. Coronado, H. S. J. van der Zant, *Adv. Mater.* **2015**, 27, 1288.
